# Cnot3 enhances human embryonic cardiomyocyte proliferation by promoting cell cycle inhibitor mRNA degradation

**DOI:** 10.1038/s41598-017-01628-0

**Published:** 2017-05-04

**Authors:** Bingying Zhou, Junwei Liu, Zongna Ren, Fang Yao, Jingwei Ma, Jiangping Song, Brian Bennett, Yisong Zhen, Li Wang, Guang Hu, Shengshou Hu

**Affiliations:** 1 State Key Laboratory of Cardiovascular Disease, Fuwai Hospital, National Center for Cardiovascular Diseases, Chinese Academy of Medical Sciences and Peking Union Medical College, Beijing, 100037 People’s Republic of China; 2 Cardiovascular Surgery, Union Hospital, Huazhong University of Science and Technology, Wuhan, Hubei 430071 People’s Republic of China; 3 Department of Immunology, Tongji Medical College, Huazhong University of Science & Technology, Wuhan, Hubei 430030 People’s Republic of China; 4 Integrative Bioinformatics, National Institute of Environmental Health Sciences, Research Triangle Park, North Carolina, 27709 USA; 5 Epigenetics and Stem Cell Biology Laboratory, National Institute of Environmental Health Sciences, Research Triangle Park, North Carolina, 27709 USA

## Abstract

Uncovering the molecular basis of mammalian cardiomyocyte proliferation may eventually lead to better approaches for heart regeneration. Compared to extensively-studied transcriptional regulation, the roles of posttranscriptional regulation in cardiac cell fate decisions remain largely unknown. Here, we identified Cnot3 as a critical regulator in cardiomyocyte proliferation at the late stage of cardiac differentiation from human ESCs. Cnot3 was highly expressed in cardiomyocytes with higher proliferation potential in both human and mouse, and its depletion resulted in significant reduction in the proliferative capacity of cells. Furthermore, Cnot3 overexpression greatly enhanced proliferation in both cultured human cardiomyocytes and infarcted murine hearts. Mechanistically, the Ccr4-Not complex preferentially interacted with anti-proliferation gene transcripts in a Cnot3-dependent manner, and promoted their degradation. Together, our study supported the model that Cnot3 enhances cardiomyocyte proliferation by promoting cell cycle inhibitor mRNA degradation. It revealed a previously unrecognized role of mRNA degradation in cardiomyocyte growth, and suggested a potential strategy to control cardiac cell fates in development and diseases.

## Introduction

Unlike invertebrates, mammalian hearts rapidly lose the capacity of regeneration after birth, resulting in limited cardiac repair in response to injury or aging. Considerable studies has been performed to explore potential targets enabling mammalian cardiomyocytes to reenter the cell-cycle, thereby effectively generating cardiomyocytes to compensate for disease-damaged myocardium. To date, transcription factors^1^, microRNAs^2, 3^, growth factors^4^ have been identified as promising targets to induce cardiomyocyte proliferation, shedding light on the regeneration of mammalian hearts. Despite these promising findings, a major hurdle in cardiac regeneration is the relative low efficiency of proliferation^5^, suggesting that other key factors involved in this process need to be determined. In addition, compared to other species, the molecular foundation of human cardiomyocyte proliferation remains largely unexplored owing to limited the availability of human hearts.

Cardiac development is a highly orchestrated process integrating a multitude of intrinsic and extrinsic signals, which together dictate the programmed transitions between distinct development stages^6–8^. Findings from heart development will not only delineate the molecular basis of organ formation, but also provide us with attractive targets to manipulate cardiac cell fates in development and diseases^9, 10^. For example, Meis1, Erbb2 and miRNA-34a are factors involved in mouse heart development and aging that also play critical roles in regulating adult cardiomyocyte proliferation^11–15^. Recently, a human pluripotent stem cell (PSC)-based cardiac differentiation model offers us with the unique opportunity to investigate the molecular foundation of human cardiac development in culture, which is complementary to our current knowledge on cardiomyocyte fate decisions learned from animal models. By use of this approach, epigenetic and genetic roadmaps controlling human cardiac development can be studied in a dish^16, 17^. However, in contrast to the extensive studies performed on transcriptional control, posttranscriptional regulation in cardiac cell fate decisions remains poorly understood.

The Ccr4-Not complex is a multisubunit complex that controls the outcome of gene expression at multiple layers, such as transcription initiation^18^, transcription elongation^19^, mRNA export^20^, RNA degradation^21^ and translation^22^. Different subunits of Ccr4-Not have been implicated in various physiological and pathophysiological processes including pluripotent stem cell fate decisions^23, 24^, somatic cell reprogramming^25^, metabolic disorders^26^, heart diseases^27^ and immune system development^28^. Therefore, it is critically important to understand the biological functions of each subunit prior to understanding the delicate control of this complex. Previous studies suggested that Cnot3, a subunit of the Ccr4-Not complex, is required for embryonic stem cell (ESC) self-renewal by inhibiting extraembryonic differentiation^23^. Interestingly, Cnot3 has also been identified as a pivotal factor in maintaining normal heart function in an RNAi screen in Drosophila^27^. Based on these observations, we set out to test whether Cnot3 plays a role in cardiac lineage commitment during ESC differentiation, and whether it therefore is a potential target of human cardiomyocyte fate manipulation.

Using a human embryonic stem cell (hESC)-based cardiac differentiation model, we found that Cnot3 preferentially binds to anti-proliferation gene transcripts, such as cyclin-dependent kinase inhibitor (CDKI) mRNAs, and that it mediates their degradation to promote robust expansion of cardiomyocytes during cardiac development. Overexpression of Cnot3 was capable of inducing cardiomyocyte proliferation in both cultured human cardiomyocytes and infarcted murine hearts. Our findings indicate an important role of Cnot3-dependent RNA degradation in the regulation of cardiomyocyte proliferation, which provides novel insights into human heart regeneration in diseases and regenerative medicine.

## Results

### Cnot3 plays an important role in hESC cardiac differentiation

**Figure 1:**
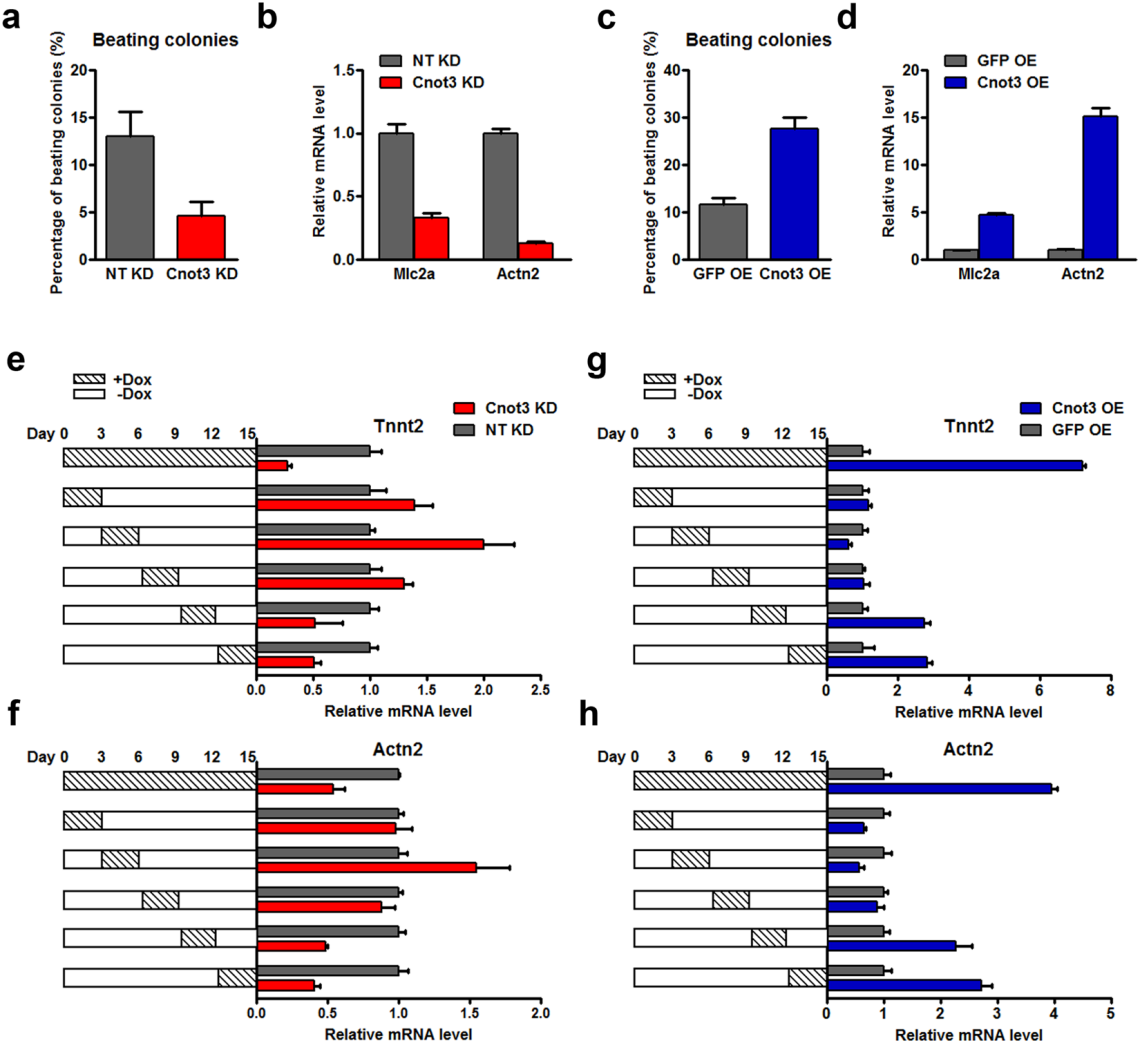
Cnot3 is required for cardiac differentiation. (**a**) NT shRNA-(shNT) or Cnot3 shRNA (shCnot3)-transduced human ESCs were induced to differentiation (see Methods for details) for 12 days, and the amount of beating colonies were counted and plotted as mean ± SEM from three independent experiments. (**b**) The expression of cardiac specific gene was determined by qPCR 12 days after hESCs differentiation. β-actin was used as endogenous control and values were plotted as mean ± SEM from three independent experiments. (**c**) GFP or Cnot3-overexpressing human ESCs were induced to differentiation (see Methods for details) for 12 days, and the amount of beating colonies were counted and plotted as mean ± SEM from three independent experiments. (**d**) The expression of myocyte specific gene was determined by qPCR 12 days after hESCs differentiation. β-actin was used as endogenous control and values were plotted as mean ± SEM from three independent experiments. (**e**,**f**) Inducible-shCnot3 or Inducible-shNT hESCs were induced to differentiation, and doxycycline was added during the differentiation process at the indicated time points. The effects of Cnot3 depletion on myocyte specific gene expression at different stages were determined by qPCR. β-actin was used as endogenous control and values were plotted as mean ± SEM from three independent experiments. (**g**,**h**) Inducible-Cnot3 or Inducible-GFP overexpression hESCs were induced to differentiation, and Doxycycline was added during the differentiation at the indicated time points. Effects of Cnot3 depletion at different stage on myocyte specific gene expression were determined by RT-qPCR. β-actin was used as endogenous control and values were plotted as mean ± SEM from three independent experiments.

To explore the role of Cnot3 in cardiac lineage commitment, we first silenced Cnot3 with a lentiviral-based shRNA during hESC cardiac differentiation via embryoid body (EB) formation. Interestingly, the percentage of beating colonies that consist of a significant number of cardiomyocytes was significantly decreased 12 days after differentiation upon Cnot3 knockdown (4.7%) compared to a non-targeting (NT) control (13.0%), *p* < 0.05 (Fig. 1a). Consistently, the expression of myocyte-specific markers, such as Mlc2a and Actn2, were dramatically suppressed upon Cnot3 depletion (Fig. 1b). On the contrary, Cnot3 overexpression in hESCs greatly promoted cardiac lineage commitment during differentiation, characterized by the increased percentage of beating colonies and elevated expression levels of cardiac lineage markers (Fig. 1c,d). In addition, we performed Fluorescence Activated Cell Sorting (FACS) to count the number of Tnnt2^+^ cardiomyocytes, and found that Cnot3 silencing led to a decreased number of cardiomyocytes whereas Cnot3 overexpression resulted in an increased number of cardiomyocytes, at day 12 during ESC random differentiation (Figure S1). To determine how Cnot3 regulates cardiac differentiation, we transduced hESC with lentivirus expressing doxycycline (Dox)-inducible NT shRNA or Cnot3 shRNA to test the requirement for Cnot3 at different stages of cardiac differentiation^29^. Red fluorescence driven by the inducible promoter confirmed the expression of shRNA vectors in our stable cell lines, and the delicate control of Cnot3 expression by Dox treatment was further verified by real time-PCR and western blotting at mRNA and protein levels, respectively (Figure S2). Intriguingly, the reduction in myocyte marker Tnnt2 and Actn2 expression was most pronounced when Cnot3 was silenced at later stages (day 9–15) of differentiation (Fig. 1e,f). Consistently, Cnot3 overexpression led to significant increases in cardiomyocyte marker expression (Fig. 1g,h). Together, these observations indicated an important role of Cnot3 in late-stage cardiac development.

### Cnot3 is required for human cardiomyocyte proliferation

**Figure 2:**
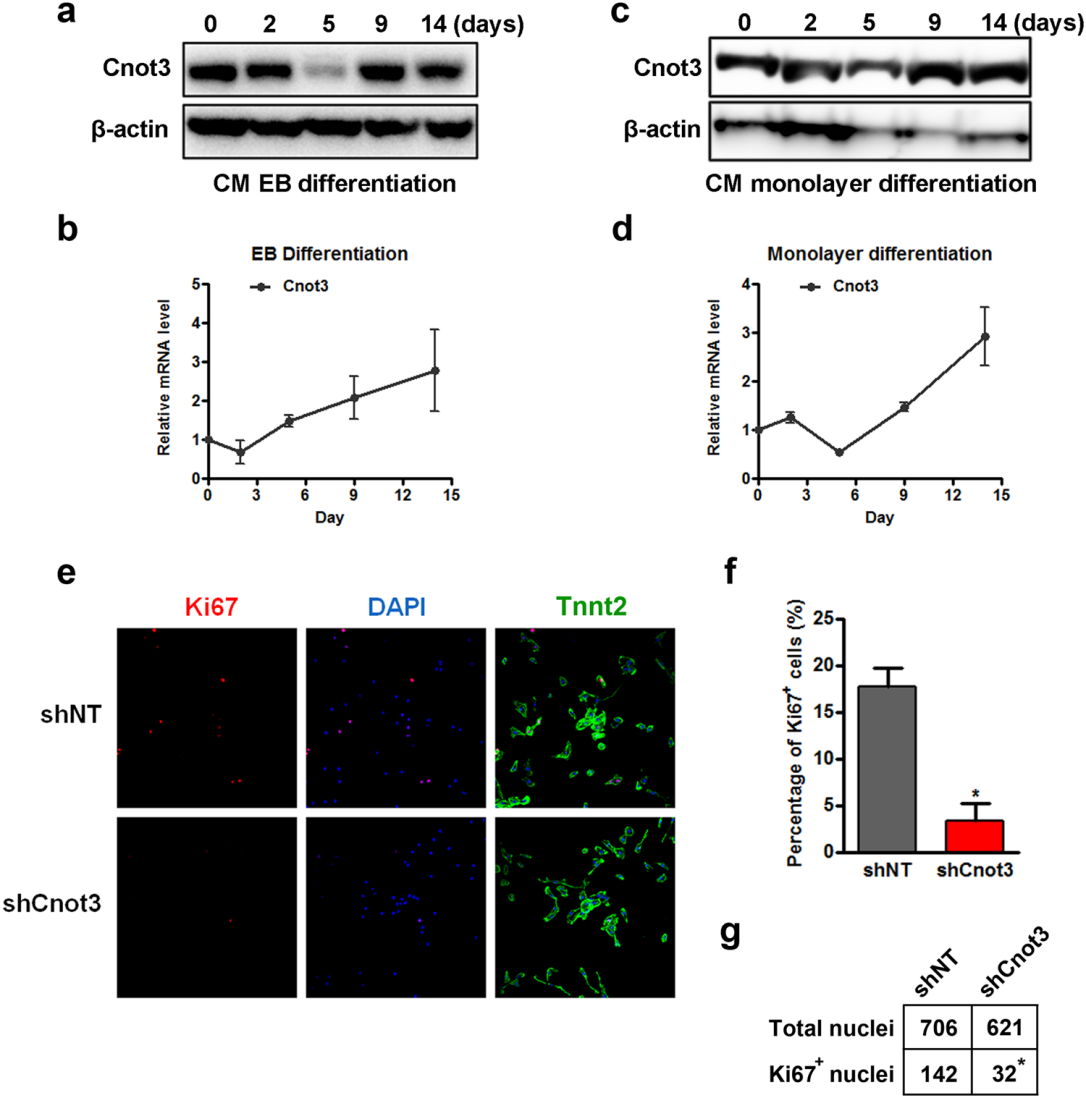
Cnot3 plays an important role in cardiomyocyte proliferation. (**a**,**b**) hESCs were induced to differentiate into cardiomyocytes by EB suspension culture. The expression of Cnot3 was determined by western blotting (**a**) and qPCR (**b**) at the indicated time points, respectively. β-actin was used as loading control in western blot and endogenous control in RT-PCR. The blots were cropped since different antibodies were incubated at the same time. (**c**,**d**) hESCs were induced to differentiate into cardiomyocytes by monolayer culture. The expression of Cnot3 was determined by both western blotting (**c**) and qPCR (**d**) at the indicated time points. β-actin was used as loading control in western blot and endogenous control in RT-PCR, respectively. (**e**) Representative images of immunofluorescence staining against Ki67 and Troponin T2 (TnnT2) to show the impacts of Cnot3 depletion on the proliferation of hESC-derived cardiomyocytes. (**f**) Ki67 positive cells were counted, and percentage of Ki67-positive cells was calculated and plotted as mean ± SEM from ten images from three independent experiments, *p < 0.05. (**g**) Ki67 positive nuclei were counted from ten images from three independent experiments, and significance was calculated by chi-square test, *p < 0.05.

To further understand how Cnot3 contributes to cardiac differentiation, we determined its expression during cardiac development using two different human ESC-based cardiac differentiation models: EB-based and monolayer-based cardiac differentiation models. Successful induction of cardiomyocytes from hESCs was confirmed by real-time PCR and immunostaining of known markers (Figure S3). In agreement with our previous results, the mRNA and protein levels of Cnot3 were increased at the late stages of cardiac development in both models (Fig. 2a,d). We first postulated that Cnot3 may directly affect key transcription factors involved in cardiac specification at the late stages of differentiation. However, no apparent changes in the expression of those factors were observed upon Cnot3 depletion (data now shown). Therefore, we hypothesized that Cnot3 has a role in cardiac cell proliferation, since cardiomyocytes experience a robust expansion at the late stage of differentiation. Indeed, immunostaining exhibited a marked reduction in Ki67-positive (Ki67^+^) cardiomyocytes when Cnot3 was depleted (*p* < 0.05) (Fig. 2e–g). Collectively, these results demonstrated a clear correlation between Cnot3 expression and cardiomyocyte proliferation.

### Cnot3 posttranscriptionally regulates cyclin-dependent kinase inhibitors (CDKIs) expression

**Figure 3:**
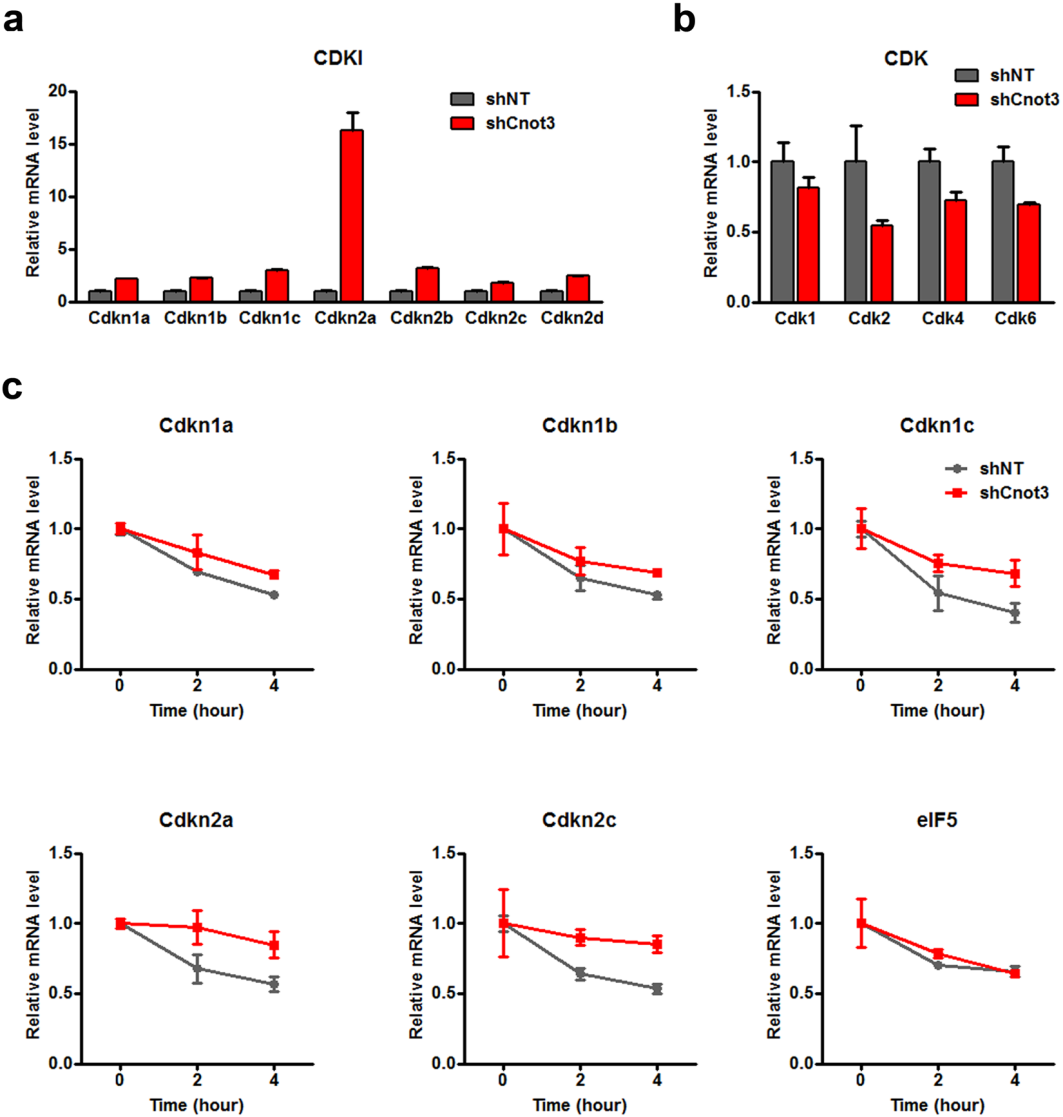
Cnot3 suppresses CDKI expression by promoting the degradation of their transcripts. (**a**,**b**) The expression of CDKI mRNA (**a**) and CDK mRNA (**b**) 96 h after Cnot3 depletion in hESC-derived cardiomyocytes was determined by qPCR. β-actin was used as endogenous control and values were plotted as mean ± SEM from three independent experiments. (**c**) hESC-derived cardiomyocytes were treated by actinomycin D (50 µg/ml), and the amounts of CDKI mRNAs upon Cnot3 or NT silencing were determined by qPCR at the indicated time points. β-actin was used as endogenous control and values were plotted as mean ± SEM from three independent experiments.

The observation that Cnot3 positively correlated with cardiomyocyte proliferation led us to hypothesize that cell cycle genes may be involved in such regulation. Therefore, we examined the expression of cyclin-dependent kinase inhibitors (CDKIs) and cyclin-dependent kinases (CDKs). We found that Cnot3 silencing led to significantly increased expression of cyclin-dependent kinase inhibitors (CDKIs), especially Cdkn2a, but only a modest decrease in CDKs (Fig. 3a,b). Importantly, neither Cnot3 silencing nor overexpression significantly altered CDKI expression in human fibroblasts, suggesting cell type specificity of Cnot3 in regulating cell cycle during cardiac development (Figure S4a,b).

Cnot3 is a subunit of the Ccr4-Not complex, which regulates gene expression by either RNA deadenylation or transcriptional regulation^24, 26^. To get a clue as to how Cnot3 exerts its effects, we determined its subcellular localization in cardiomyocytes via immunostaining. Using a validated antibody against Cnot3, we found that Cnot3 was predominantly distributed in the cytoplasm, suggesting a possible role of Cnot3 in the direct regulation of target gene mRNAs (Figure S4c). Given that RNA deadenylation often leads to the accelerated degradation of target mRNAs, we proposed that Cnot3 may inhibit CDKI expression by promoting the decay of their transcripts. To test our hypothesis, we applied actinomycin D to inhibit transcription globally, and observed the impact of Cnot3 on the stability of CDKI transcripts. Consistent with the changes in mRNA expression (Fig. 3a), Cnot3 depletion significantly reduced the degradation of CDKI mRNAs, but not that of eIF5, a protein involved in protein translation (Fig. 3c). Together, these results indicated that Cnot3 promotes proliferation of cardiomyocytes by facilitating the degradation of the mRNAs of CDKIs.

### Ccr4-Not preferentially interacts with anti-proliferation gene transcripts in a Cnot3-dependent manner

**Figure 4:**
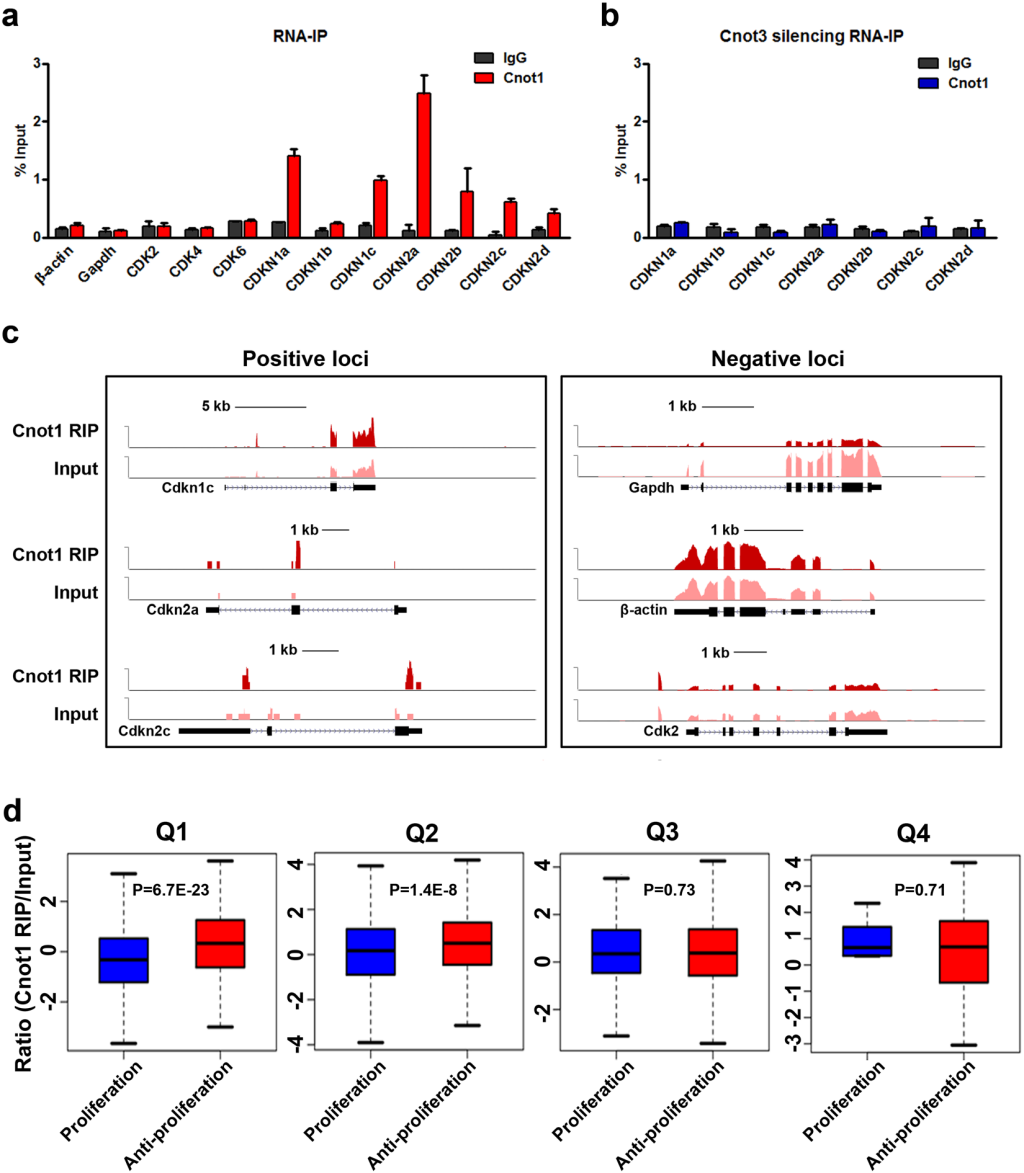
Cnot1 preferentially interacts with anti-proliferative gene transcripts in a Cnot3-dependent manner. **(a)** Cnot1 interaction with mRNAs. RIP was performed in hESC-derived cardiomyocytes with either IgG or Cnot1 antibody. Relative abundance of the immunoprecipitated mRNAs was determined by RT-qPCR, normalized to Input, and plotted as mean ± SEM from three independent experiments. (**b**) Effects of Cnot3 silencing on Cnot1 RIP. hESC-derived cardiomyocytes were transduced with shCnot3, and RIP was conducted 48 h after transduction. Values were plotted as mean ± SEM from three independent experiments. (**c**) Cnot1 RIP sequencing. Cnot1 RIP was performed in hESC-derived cardiomyocytes, and the total and Cnot1-interacting RNAs were subjected to high-throughput sequencing. Representative images form the genome browser were shown for the indicated genes. For each gene, upper track: Cnot1 RIP; lower track: Input. (**d**) Statistical analysis of Cnot1 RIP-sequencing. Genes detected in the sequencing were ranked by their expression level in hESC-derived cardiomyocytes and divided into four equal groups (Q1–Q4). Cnot1 binding was determined by the ratio of the reads per kilo base per million (RPKM) in Cnot1 RIP/Input and compared between Proliferative and Anti-proliferative genes in each quarter. Proliferative genes were defined as those downregulated in long-term cultured hESC-CMs, while Anti-proliferative genes were defined as those upregulated (See Experimental Procedures for details). *P* values were calculated by the Wilcoxon rank-sum test.

To further understand the mechanism by which Cnot3 affects the mRNA stability of those genes, we asked whether Cnot3 regulates them via direct binding. To this end, we carried out RNA immunoprecipitation (RNA-IP) against Cnot1, a scaffold and key component in the Ccr4-Not complex that binds mRNAs, to test the binding activity of this complex on different mRNAs. In contrast to housekeeping gene mRNAs (β-actin and GAPDH), CDKI mRNAs were preferentially bound by Cnot1 (Fig. 4a). More importantly, the binding activity of Cnot1 on those mRNAs was markedly compromised upon Cnot3 silencing, indicating a crucial role of Cnot3 in mediating the interaction between Ccr4-Not and CDKI mRNAs (Fig. 4b).

To globally test whether Ccr4-Not preferentially interacts with a subset of anti-proliferation gene mRNAs, we conducted RNA-IP followed by high-throughput sequencing (RIP-Seq) for Cnot1 14 days after hESC-cardiac differentiation. Consistent with the above real time PCR results, genome browser tracks showed that significant amounts of mRNA from CDKIs such as Cdkn1a, Cdkn2a and Cdkn2c, were recovered in the Cnot1 RIP sample compared to the Input, whereas other mRNAs including Gapdh, β-actin and Cdk2 were not enriched (Fig. 4c). More importantly, there was a significant increase in Cnot1 binding with transcripts that are associated with human cardiomyocyte proliferation in general, as the ratio of reads per kilobase per million (RPKM) of Cnot1 RIP/Input was higher for anti-proliferation versus proliferation gene mRNAs in abundantly expressed gene population (Q1–Q2, Fig. 4d). Together, our data suggest that Ccr4-Not preferentially interacts with anti-proliferation gene mRNAs and facilitates their degradation to promote human cardiomyocyte proliferation at the late stage of cardiac differentiation.

### Cnot3 overexpression enhances human cardiomyocyte proliferation

**Figure 5:**
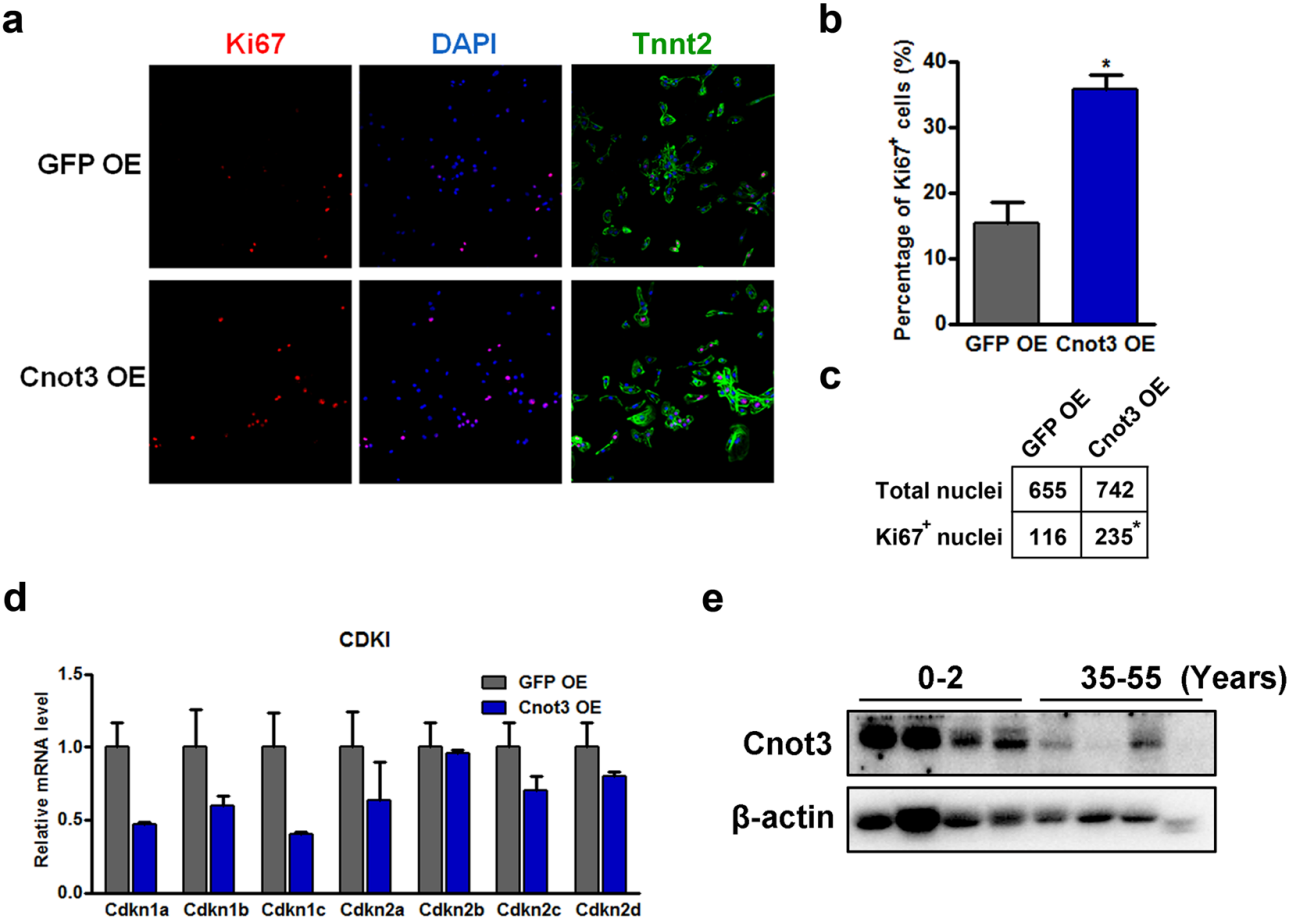
Cnot3 overexpression induces human cardiomyocyte proliferation. (**a**) Representative images of immunofluorescence staining against Ki67 and Troponin T2 (TnnT2) to show the impacts of Cnot3 overexpression on the proliferation of hESC-derived cardiomyocytes. (**b**) Percentage of Ki67 positive cells was counted, and plotted as mean ± SEM from ten images from three independent experiments, *p < 0.05. **(c)** Ki67 positive nuclei were counted from ten images from three independent experiments, and significance was calculated by chi-square test, *p < 0.05. (**d**) qPCR to show the expression of CDKI mRNA 96 hours after Cnot3 overexpression in hESC-derived cardiomyocytes. β-actin was used as endogenous control and values were plotted as mean ± SEM from three independent experiments. **(e)** Western blot to show the Cnot3 protein levels in human heart tissues from adult versus infant. β-actin was used as loading control. The blots were cropped since different antibodies were incubated at the same time.

Given the proliferative effects of Cnot3 during cardiac differentiation, we asked whether Cnot3 overexpression could induce human cardiomyocyte proliferation. Intriguingly, Cnot3 overexpression greatly increased the percentage of Ki67-positive cells in human cardiomyocytes compared to GFP overexpression, *p* < 0.05 (35.7% vs. 15.3%, Fig. 5a–c). In accordance with the previous findings, ectopic Cnot3 expression suppressed CDKIs expression (Fig. 5d). Since previous findings suggested a relative higher turnover rate of cardiomyocytes in neonatal hearts compared to adult hearts, we examined whether Cnot3 expression correlated with cardiomyocyte proliferation in human hearts. Strikingly, Cnot3 was more robustly expressed in human infant hearts compared to adult hearts (Fig. 5e), which suggests a promising role of Cnot3 in regulating cardiomyocytes proliferation in development and aging.

### Cnot3 overexpression promotes cardiomyocyte proliferation *in vivo*

**Figure 6:**
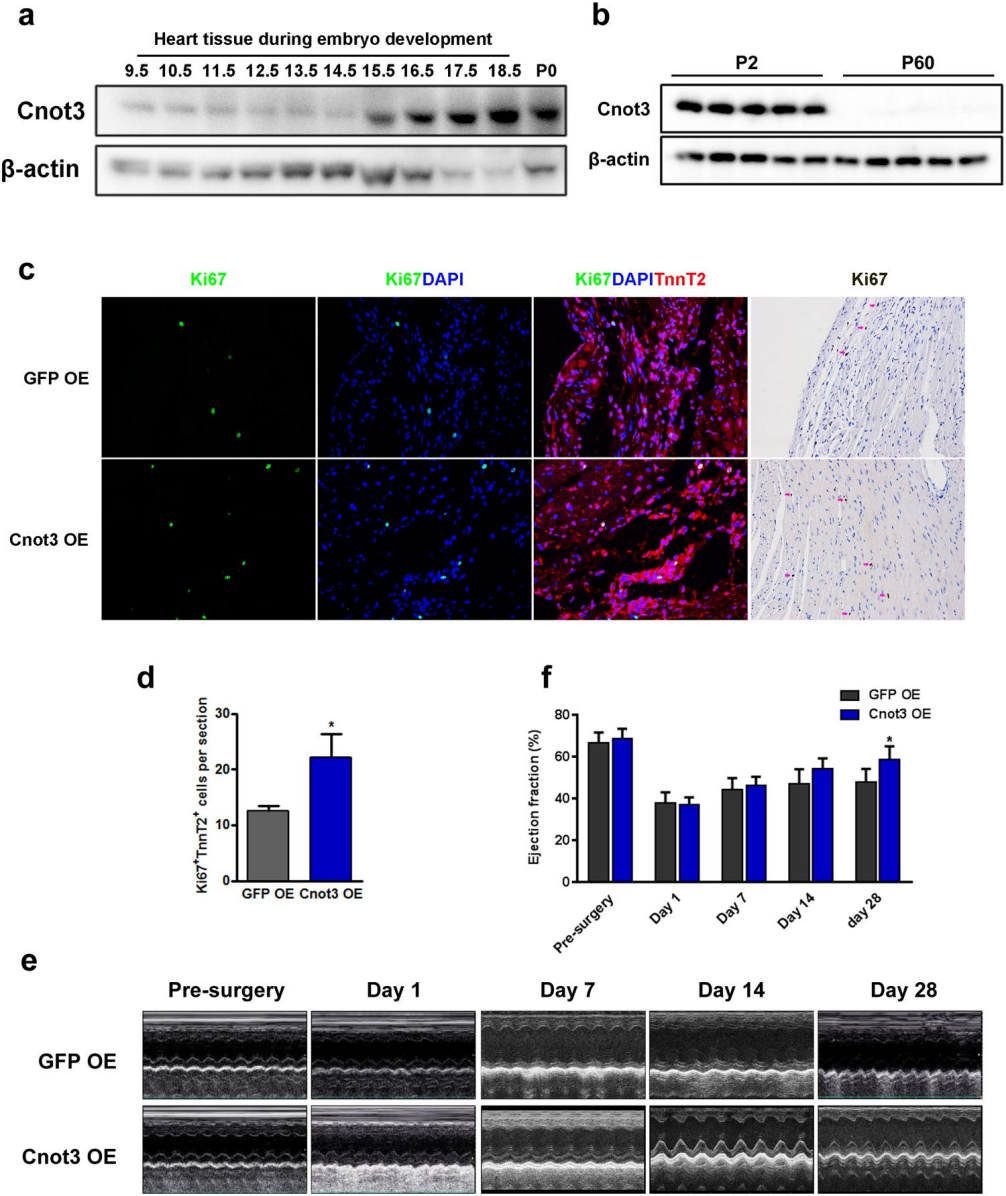
Cnot3 plays a critical role in cardiomyocyte proliferation *in vivo*. (**a**) Western blots to show Cnot3 protein level in developing hearts from mouse embryos at indicated time points. β-actin was used as loading control. The blots were cropped since different antibodies were incubated at the same time. (**b**) Western blot to show Cnot3 protein level in mouse neonatal hearts versus adult hearts. β-actin was used as loading control. The blots were cropped since different antibodies were incubated at the same time. (**c**) Representative images of immunofluorescence or immunochemical staining against Ki67 to show the impact of Cnot3 overexpression on cardiomyocyte proliferation in a mouse myocardial infarction model (See experimental procedures for details). DAPI (blue) was used to stain the cell nuclei. Magnification: 100x. (**d**) Statistical analysis of (C). Ki67^+^TnnT2^+^ cells were counted from 10 sections per mouse, 6 mice per group, values were plotted as mean ± SEM from three independent experiments, *p < 0.05. (**e**) Representative images of echo to show heart functions in GFP overexpression or Cnot3 overexpression hearts at indicated time points, respectively. (**f**) Statistical analysis of (**e**). Values were calculated as fractional shortening, and plotted as mean ± SEM. n = 6 per group, *p < 0.05.

To determine whether Cnot3 has a similar effect on proliferation *in vivo*, we first examined the expression of Cnot3 in developing mouse hearts. Cnot3 protein expression was significantly increased at the late stage of heart development (Fig. 6a). We also assessed Cnot3 expression in neonatal mouse heart versus adult mouse heart, and found that, strikingly, Cnot3 abundantly expressed in neonatal (P2) mouse heart, but was nearly undetectable in adult mouse heart (P60) (Fig. 6b). This observation was consistent with previous findings that mouse heart experiences a rapid loss of proliferation capacity after birth. It is also consistent with our findings in human heart samples, and strongly suggests a possible role of Cnot3 in cardiomyocyte proliferation *in vivo*. To test that, we injected lentivirus overexpressing Cnot3 into mouse hearts immediately after myocardial infarction surgery and evaluated local cardiomyocyte proliferation 21 days post-surgery. Intriguingly, after validation of successful overexpression of Cnot3 in the myocardium (Figure S5a,b), we found that Cnot3 overexpression significantly enhanced local cardiomyocyte proliferation compared to GFP overexpression, as characterized by a nearly 2-fold increase in Ki67-positive cardiomyocytes in the myocardium (Fig. 6c,d). In addition, Cnot3 overexpression slightly repressed CDKI expression while promoting CDK expression in infarcted hearts (Figure S5c,d). Consistently, cardiac fractional shortening and ventricular thickness were improved with Cnot3 overexpression (Fig. 6e,f). Collectively, these results indicate that Cnot3 manipulation is an effective means to enhance cardiomyocyte proliferation and improve heart function in heart diseases.

## Discussion

Although extensive efforts have been made to uncover the molecular basis of cardiac cell fate decisions, the inefficient proliferation of cardiomyocyte remains a major obstacle limiting myocardial regeneration in potential clinical applications. Using a hESC differentiation model, our study reveals that Cnot3 plays an essential role in the expansion of cardiomyocytes at the late stage of human cardiac development. Cnot3 overexpression promotes cardiomyocyte proliferation in both human cardiomyocytes and infarcted mouse myocardium. Therefore, manipulation of Cnot3 provides us with a potential strategy to enhance myocardial regeneration and heart function in diseases.

### Embryonic stem cell cardiac differentiation as a model to study human heart development

ESCs are derived from the inner cell mass of blastocysts. They can be maintained indefinitely while retaining the potential to differentiate into any cell type from the three germ layers. Thus, ESC differentiation offers us the unique opportunity to investigate mammalian development, especially human development, in a dish. Prior to isolation of human ESCs, most heart development studies were carried out in animals, such as mice and zebrafish. Although these studies greatly enhanced our understanding of the molecular foundation of heart development, we cannot apply those findings directly in the clinical applications due to physiological differences among species. Using a hESC differentiation system, we found that Cnot3 plays a critical role in the proliferation of human cardiomyocyte. Taking the advantage of this *in vitro* differentiation system, we uncovered that Cnot3 preferentially interacts with anti-proliferation gene transcripts and mediates their degradation. Given the immature properties of ESC-derived cardiomyocytes, further verification of the effects of Cnot3 on cardiomyocyte proliferation may be required in mature human cells.

### Posttranscriptional regulation in cardiomyocyte fate decisions

Regulation of gene expression occurs at multiple levels to form delicate networks controlling cell fate decisions. However, compared to the well-characterized transcriptional regulation in heart development, posttranscriptional regulation in cardiac cell fate decisions is less clear. Growing studies suggested that microRNA and long noncoding RNA play crucial roles in cardiomyocyte proliferation^2^, cardiac dilation and dysfunction^30^, embryonic heart development^31, 32^, and cardiac hypertrophy^33^, underscoring the importance of posttranscriptional regulation in cardiac cells. In addition, a recent study also reported that RNA binding protein Trbp-mediated microRNA processing is required for normal heart function^34^, highlighting the delicate regulatory networks occurring at the posttranscriptional level to control cell fates. Consistent with this idea, we found that the Ccr-Not4 complex preferentially binds to a group of anti-proliferation gene transcripts and mediates their decay in cardiomyocytes. Interestingly, our findings experimentally support the “RNA regulon” concept that functionally associated RNAs are coordinately regulated by specific mRNP processing machineries at various steps after transcription, such as mRNA export^35, 36^. By mediating the degradation of a group of anti-proliferation gene transcripts, we propose that Ccr4-Not4 complex is an efficient hub integrating proliferation and maturation signals to ensure coordinated transitions between different developmental stages. It would be therefore interesting to explore how cardiac cell fates are posttranscriptionally controlled at other steps.

### The potential role of Cnot3 in cardiac diseases and maturation

Many key factors identified to play critical roles in development are also important players in heart diseases. For example, Brg1 epigenetically regulates cardiac growth, differentiation and gene expression in developing hearts, and thereby maintains cardiomyocytes in an embryonic state. The re-expression of Brg1 in adult mouse underlies cardiac hypertrophy^37^. Similarly, our study suggests a critical role of Cnot3 in the expansion of cardiomyocytes at the late stage of cardiac development, and that the expression of Cnot3 is almost undetectable in adult heart while abundantly expressed in neonatal hearts. Thus, it will be interesting to explore whether ectopically expressed Cnot3 in adult hearts underlies cardiac diseases. On the other hand, human pluripotent stem cell (hPSC)-derived cardiomyocytes are immature cells, exhibiting molecular and functional features resembling embryonic cardiomyocytes, which hampers the applications of stem cell therapy in heart regeneration. Given the rapid diminishment of Cnot3 expression after birth, it is therefore possible that depletion of Cnot3 may accelerate the maturation of hESC-derived cardiomyocyte. Interestingly, a previous study revealed that heterozygous Cnot3 knockout mice displayed impaired cardiac contractility and increased susceptibility to heart failure, further indicating the multiple roles of Cnot3 in cardiac cell fate decisions^27^.

### Ccr4-Not complex in development and diseases

Our data suggest that Ccr4-Not complex preferentially interacts with a group of genes involved in cardiomyocyte proliferation in a Cnot3-dependent manner. In contrast to cardiomyocytes, Cnot3 depletion failed to increase CDKI expression in fibroblast, suggesting tissue specificity of Cnot3 in regulating proliferation. In addition, Cnot3 has also been reported to play important roles in embryonic stem cell self-renewal and somatic cell reprogramming in mice^23–25^. Thus, it is important to delineate how Cnot3 recognizes and regulates distinct subsets of gene expression in different contexts. According to previous studies, different components in this complex have been implicated in different biological behaviors. For example, Cnot7 knockout mice displayed severe infertility^38^, whereas Cnot6 deficient mice were viable and fertile^39^. Together, these observations suggest that the Ccr4-Not complex may utilize different combinations of its subunits to execute distinct functions.

Given that Ccr4-Not regulates gene expression at multiple steps, how Cnot3 mediates mRNA degradation remains an open question. Previous studies indicated that Cnot3 is capable of regulating gene expression via mRNA deadenylation^26^. In addition, the Ccr4-Not complex has been reported to degrade RNA via miRNA-GW182-RISC^40^. Moreover, our recent work identified that Cnot3 safeguards ESC self-renewal via degrading differentiation gene transcripts, consistent with our findings that Cnot3 regulates cardiac cell fates posttranscriptionally^41^. Further experiments are needed to determine whether Cnot3 promotes CDKI mRNA deadenylation and subsequent degradation in the context of our study. Moreover, it is particularly interesting that Cnot3 exhibits high preference for binding a subset of mRNAs instead of all mRNAs. Understanding the mechanism of this biased binding of Cnot3 will provide important indications for the delicate regulation of function-related gene subsets in development and diseases.

## Methods

### Human Embryonic Stem (ES) Cell Culture and Differentiation

Human H1 ES cells were purchased from and maintained on Matrigel-coated tissue culture plates with E8 medium (Stemcell Technologies) as previously described, and the medium was changed on a daily basis. Cells were passaged by ethylenediamine tetraacetic acid (EDTA, 0.5 mmol/L) detachment when reached 80% confluency. Briefly, cells were washed once with PBS, and incubated with EDTA for 5 minutes at 37 °C. Then, EDTA was removed, and cells were washed off with E8 medium followed by a brief spin down. Afterwards, the cells were re-suspended with E8 medium and subpassaged at a ratio of 1:5~1:10 in medium supplemented with a ROCK inhibitor (10 µmol/L, SelleckChem).

For ES cell random differentiation, H1 cells were dissociated by EDTA and counted as single cells. Then 3 × 10^5^ cells were plated into 1 well of a 24-well low attachment culture plate (Corning) in differentiation medium (DMEM/F12 medium containing 20% knockout serum replacement (KSR, Invitrogen), 2 mmol/L L-glutamine, 1 × nonessential amino acids (NEAA, Invitrogen), 0.1 mmol/L 2-mercaptoethanol (Invitrogen), and 1 × antibiotics (Invitrogen)) with ROCK inhibitor (10 µmol/L). The medium was changed every other day to allow efficient embryonic body (EB) formation. Six days after suspension culture, EBs were seeded onto 0.1% gelatin-coated plates and maintained in the same medium for an additional 6 days. Cells were then harvested for quantification of cardiac specific gene expression or subjected to immunostaining.

For ES cell-cardiac differentiation, 2.5 × 105 H1 cells were plated into 1 well of Matrigel-coated 6-well plates, and cultured in E8 medium for 3 days. The medium was then replaced with cardiac differentiation basal medium 1 (RPMI1640 containing 1 × B27 minus insulin supplement) containing 3 µM GSK3 inhibitor ChIR (SelleckChem) when cells were nearly 100% confluent. After 2 days of incubation, the medium was again changed to basal medium 1 for another day. Then, the cells were incubated in basal medium 1 with 5 µM Wnt inhibitor IWR-1 (SelleckChem) for 2 days followed by a 3 day-incubation with basal medium 1 only. Finally, the medium was replaced with cardiac differentiation basal medium 2 (RPMI1640 containing 1 × B27 supplement) for long-term culture. Medium was changed every 3 days until cells were harvested at desired time points.

### Human samples

Approval for this study was obtained from the Ethics committee of Union Hospital, Tongji Medical College, Huazhong University of Science and Technology. All methods were performed in accordance with the relevant guidelines and regulations. All study samples were heart atrial tissues obtained from donors of heart transplantation at Union Hospital (Wuhan, China), with written informed consent from the patients.

### Western blotting

CD1 mouse embryos were surgically collected from the uterus at the indicated time points, and hearts were dissected under the microscope. The dissected hearts were washed twice with ice-cold PBS and lysed in cell lysis buffer containing 1 × protease inhibitor (Roche), followed by homogenization. Cultured cells were washed twice with ice-cold PBS, and were directly lysed in cell lysis buffer. Human heart tissues were flash frozen in liquid nitrogen, and then homogenized into small pieces in liquid nitrogen. Tissue pieces were lysed in lysis buffer and sonicated. All lysates were subsequently sonicated and quantified by BCA assay (Life Technologies). Forty micrograms of protein was loaded and separated by SDS-PAGE on 4–12% Bis-Tris gels (Life Technologies) and transferred to nitrocellulose membranes. Membranes were blocked with 5% milk in TBS-T and then incubated with primary antibodies overnight at 4 °C. The next day, membranes were incubated with the appropriate secondary antibodies and developed using ECL Detection Kit (GE Healthcare Bio-Sciences).

### RNA isolation, reverse transcription, and RT-qPCR

Total RNA was isolated from cells using the GeneJet RNA purification kit (Thermo Scientific), and 0.5 μg total RNA was reverse transcribed to generate cDNA using the iScript cDNA Synthesis Kit (Bio-Rad) according to manufacturer’s instructions. qPCR was performed using the SsoFast EvaGreen Supermix (Bio-Rad) on the Bio-Rad CFX-384 or CFX-96 real-time PCR System. Actin was used for normalization. Primers used in the study are listed in Table S1.

### Plasmid construction

GIPZ human Cnot3 shRNAs were purchased from Open Biosystems. After verification of knockdown efficiencies of all hairpins, those with greatest efficiencies were used for the generation of pHAGE-Mir vector-based constitutive or inducible shRNA plasmids. For constitutive shRNA plasmids, both GIPZ-shCnot3 and pHAGE-Mir-Phes were digested by EcoRI and ApaI. For inducible plasmids, both GIPZ-shCnot3 and pHAGE-Mir-Phes were digested by EcoRI and MluI. The digested products were extracted and purified by a Gel purification kit (Qiagen). Then, the purified Cnot3 shRNA and Phage-Mir vector was ligated at a ratio of 3:1 with the T4 ligation system at 16 °C overnight. Afterwards, the ligation product was transformed, and amplified for the use in the future.

To generate Cnot3 overexpression plasmids, the coding region of human Cnot3 was amplified by RT-PCR and cloned into pHAGE-EF1α or pHAGE-Inducible destination expression vectors using Gateway cloning. The cloned human Cnot3 fragment was sequenced, and the expression of Cnot3 was verified in subsequent experiments.

### Lentivirus production and infection

293 T cells were plated at 6.5 × 105 cells per well in 6-well plate and cultured overnight. The culture medium was replaced with fresh medium 6 h prior to transfection. Cells were transfected with 2 µg target plasmid along with 2 µg packaging plasmids (the ratio of psPAX2 and pMD2.G is 2:1) by Lipofectamine 2000 (Invitrogen) according to the manufacturer’s instructions. The medium was changed 6–16 hours after transfection, and viral supernatant was collected 48 hours after transfection. The collected supernatant was spun down at maximum speed, filtered through a 0.45 µm filter (Millipore), and stored at −80 °C.

### Stable ES cell line generation

To generate inducible human ES cell lines, 1 × 105 cells were plated into 1 well of 12-well plate and infected with 100 µl lentivirus carrying Cnot3 shRNA or Cnot3 overexpression plasmids. Twenty four hours post infection, cells were treated with puromycin (1 µg/ml) for another 48 h. After puromycin selection, cells were expanded, and the inducibility of constructs was verified by qPCR or western blotting in the presence of doxycycline (1 µg/ml).

### Fluorescence-activated cell sorting (FACS)

Randomly differentiated EBs were collected at day 12, washed twice with PBS, digested with 0.25% trypsin at 37 °C for 5 minutes, and then dispersed into single cells by brief vortexing. The digested single cells were neutralized with 50% FBS in PBS, and washed twice with PBS. Afterwards, the cells were fixed with 1% paraformaldehyde for 10 minutes in 37 °C water bath, permeabilized with ice-cold 90% methanol for 30 minutes, and incubated with pre-diluted Tnnt2 antibody overnight at 4 °C. Meanwhile, cells incubated with normal IgG were served as negative control during sorting. After primary antibody incubation, the cells were incubated with fluorescence-conjugated secondary antibody for 30 minutes at room temperature in the dark. Then the cells were washed and dispersed in FACS buffer (PBS (w/o Ca/Mg^++^) +2% FBS + 0.1% NaN_3_) for cell sorting. BD FACSAria II (BD Biosciences) was employed in our experiments. The percentage of Tnnt2^+^ cells were calculated from 3 independent experiments.

### Immunofluorescence staining

Cells were fixed with 4% paraformaldehyde, permeabilized with 0.1% Triton X-100 in PBS, and blocked with 0.5% BSA/PBS. They were then incubated with the primary antibodies Actinin 2 (1:100, Abcam), Troponin T2 (1:100, Bethyl), Cnot3 or Ki67 at 4 °C overnight. The next day, cells were incubated with the appropriate secondary antibodies (Life Technologies). Nuclei were counterstained with DAPI (Invitrogen), and confocal images were taken on the Zeiss LSM 710 microscope.

For mouse heart staining, mouse hearts were removed, fixed with 4% paraformaldehyde and then embedded in Optimal Cutting Temperature compound (OCT). Cryosections (7 µm thickness, 350 µm apart) were taken from the middle portion of infarcted myocardium, and 8 slices of each sample were analyzed by immunostaining.

### RNA immunoprecipitation, sequencing and analysis

RNA immunoprecipitation (RIP) was carried out using the Magna RIPTM RNA-Binding Protein Immunoprecipitation Kit (Millipore) according to manufacturer’s instructions. The eluted RNAs were precipitated, quantified, and subjected to qPCR or library generation for sequencing. RNA sequencing libraries were prepared with the TruSeqTM RNA Sample Preparation Kit (Illumina). The resulting libraries were sequenced on the NextSeq 500 (Illumina) and the sequencing results will be submitted to the GEO database.

Reads from both the Cnot3 RIP and Input samples were aligned using Bowtie 0.12.8 to an index of RefSeq transcripts. Up to ten alignments per read were allowed, with up to two mismatches. RPKM values were then calculated for each RefSeq transcript. For Fig. 4d, 10,082 genes with sufficient sequencing coverage (total mapped reads > 5, RPKM > 1) were used for the analysis. They were grouped into four equal quartiles based on their input expression level: Q1 (Low expression), Q2 (Medium-low expression), Q3 (High-medium expression), and Q4 (High expression). In each quartile, the RPKM ratio of Thoc2 RIP/Input for each ESC and non-ESC gene was calculated, and Wilcoxon rank-sum test was applied to assess the binding preference of Cnot3 between proliferative and anti-proliferative genes, and the p values were then adjusted using Benjamini Hochberg method. Proliferation genes were defined as those showing at least 2-fold upregulation in 1-month hESC-derived cardiomyocyte compared 6-month hESC-derived cardiomyocyte. In contrast, anti-proliferative genes were defined as those showing at least 2-fold upregulation in 6-month versus 1-month hESC-CMs. The RNA-Seq data of 1-month and 6-month hESC-CMs will be submitted to GEO database.

### Myocardial infarction model and lentivirus injection

All animal protocols were approved by the Animal Care and Use Committee of Union Hospital, Tongji Medical College, Huazhong University of Science and Technology. All methods were performed in accordance with the relevant guidelines and regulations. Acute myocardial infarction (AMI) was created in 10–12 week old male C57/B6 mice by permanent ligation of the left anterior descending coronary artery (LAD). Animals were intraperitoneally anesthetized with sodium pentobarbital (50 mg/kg) and mechanically ventilated with room air by using Minivent 845 (Hugo Sachs Electronics, March, Germany). The heart was exposed through a left-sided minithoracotomy, and the left coronary artery was permanently ligated. Infarction was visually confirmed by observation of blanching of the left ventricular myocardium as well as dyskinesis. Immediately after LAD ligation, the mice were randomly allocated to receive intramyocardial injections of GFP- or Cnot3-overexpression lentivirus at three sites in the infarct border zone.

### Echocardiography study

For echocardiography study, mice were anesthetized by 2% isoflurane with oxygen (0.8 l/min). Mouse echocardiography was carried out by use of an echocardiograph (Philips) at indicated time points. Ejection Fraction (EF) was employed to evaluate heart function of mouse.

### Statistical analysis

Statistical significance was evaluated by paired or unpaired Student’s *t* test (two-tail) for comparison between two groups without specific description. *P < 0.05 was considered statistically significant.

## Electronic supplementary material

Supplementary Info
