# Supplementary material for: Cnot3 enhances human embryonic cardiomyocyte proliferation by promoting cell cycle inhibitor mRNA degradation
Source: Sci Rep. 2017 May 4;7:1500. doi: 10.1038/s41598-017-01628-0 (PMC5431451; doi:10.1038/s41598-017-01628-0)
Supplement: Supplementary file 1 — Supplementary Info [file 41598_2017_1628_MOESM1_ESM.pdf]

Cnot3 enhances human embryonic cardiomyocyte proliferation by promoting cell cycle inhibitor mRNA degradation

Bingying Zhou[1#], Junwei Liu[2#], Zongna Ren[1], Fang Yao[1], Jingwei Ma[3], Jiangping Song[1], Brian Bennett[4], Yisong Zhen[1], Li Wang[1*], Guang Hu[5*], Shengshou Hu[1*]

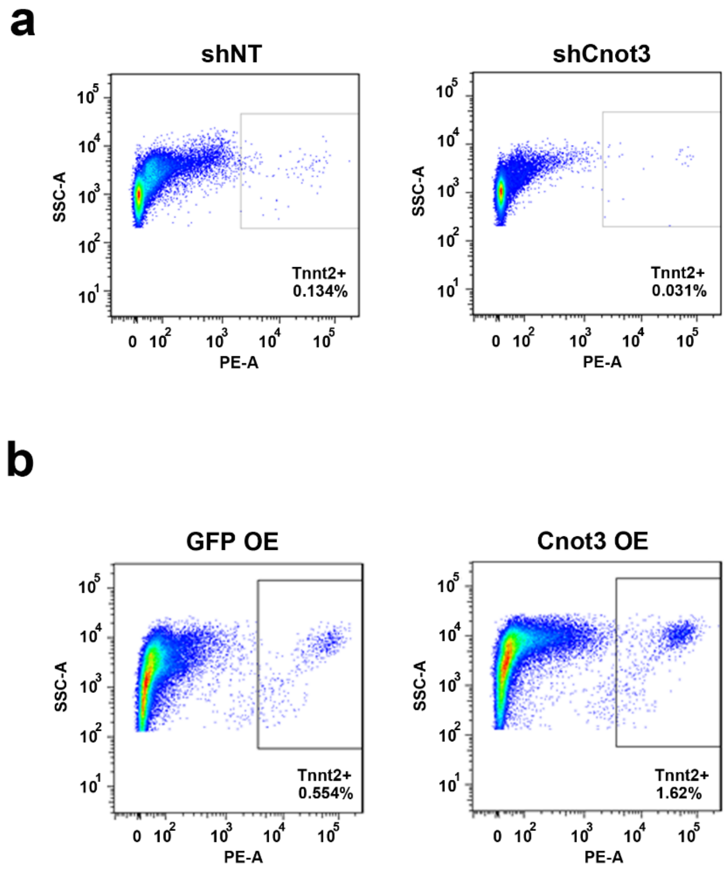

Figure S1

Figure S1. Cnot3 is required for cardiac lineage commitment. (a-b) FACS to show Tnnt2-positive ($Tnnt2^+$) cells upon Cnot3 silencing (a) or Cnot3 overexpression (b) 12 days after ESC random differentiation. The percentage of $Tnnt2^+$ cells were calculated from 3 independent experiments.

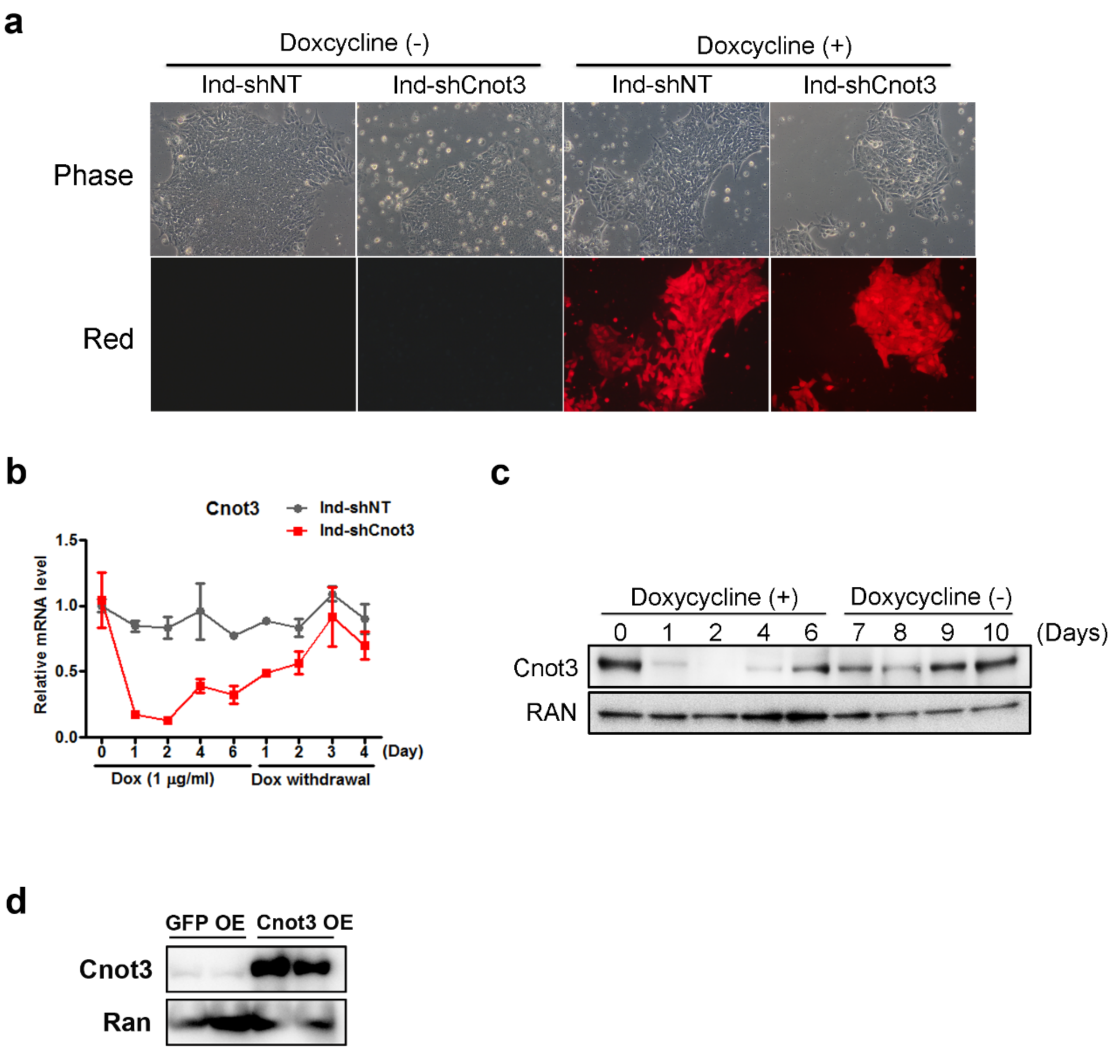

Figure S2

Figure S2. Generation of inducible hES cell lines. (a) Representative images to show the inducibility of Cnot3 shRNA or NT shRNA in the presence of Doxycycline (Dox, 1 μg/ml). **(b)** RT-PCR to show the knockdown efficiency of Cnot3 in the absence or presence of Dox. β-actin was used as endogenous control. **(c)** Western blot to show the knockdown efficiency of Cnot3 in the absence or presence of Dox. Ran was used as loading control. **(d)** Western blot to show the overexpression of Cnot3 in the presence of Dox. Ran was used as loading control.

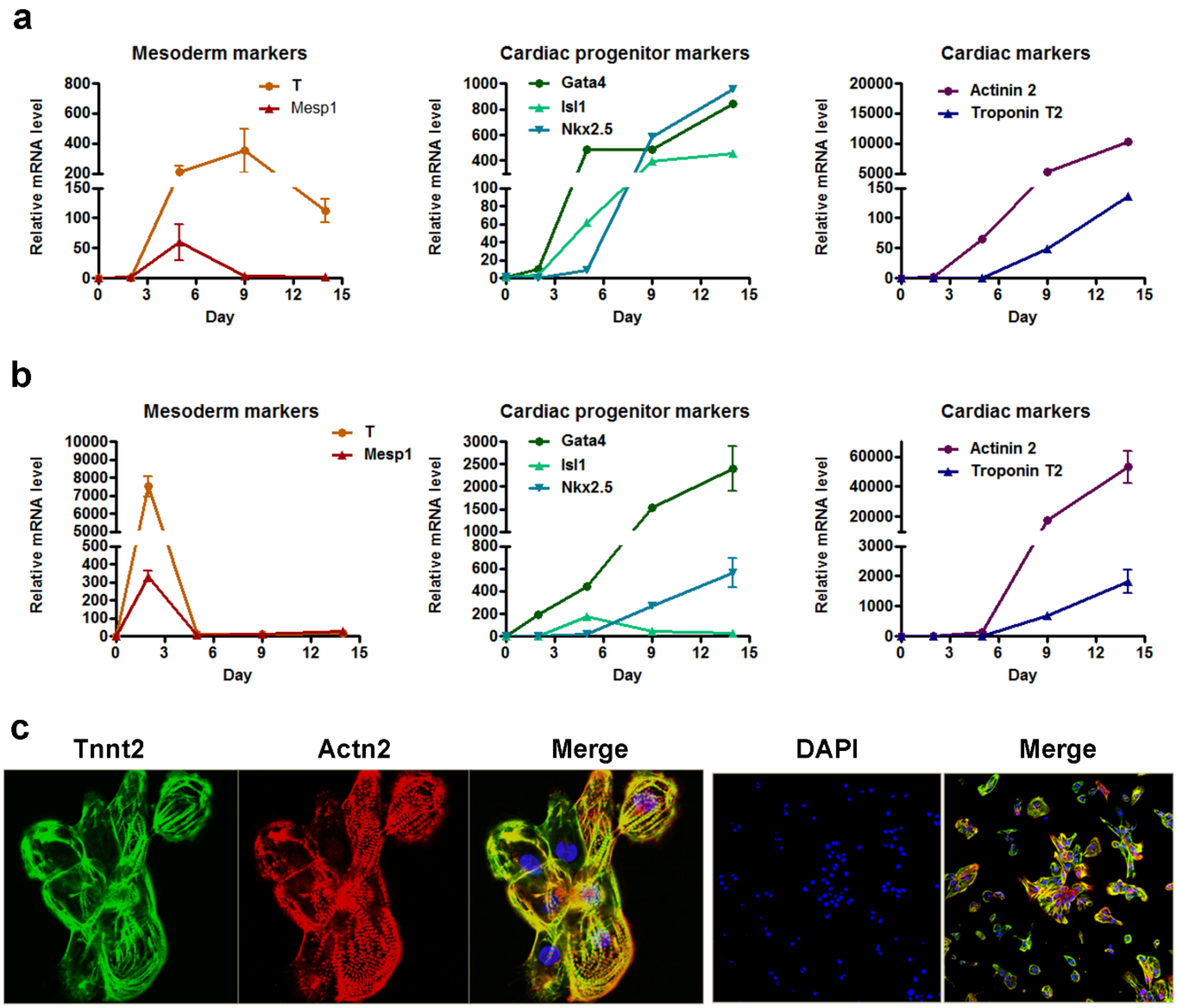

Figure S3

Figure S3. Cardiomyocyte differentiation from hESC recapitulates the molecular signature of heart development. (a-b) RT-PCR to show the expression of stage-specific markers at indicated time points during hESC-cardiac differentiation by EB suspension (a) or monolayer (b), respectively. β-actin was used as endogenous control, and values were plotted as mean ± SEM from three independent experiments. **(c)** Representative immunofluorescence staining against Tnnt2 (Green) and Actn2 (Red) to show the structure and purification of induced cardiomyocytes. DAPI (blue) was used to stain the cell nuclei. Magnification: 630× and 100×, respectively.

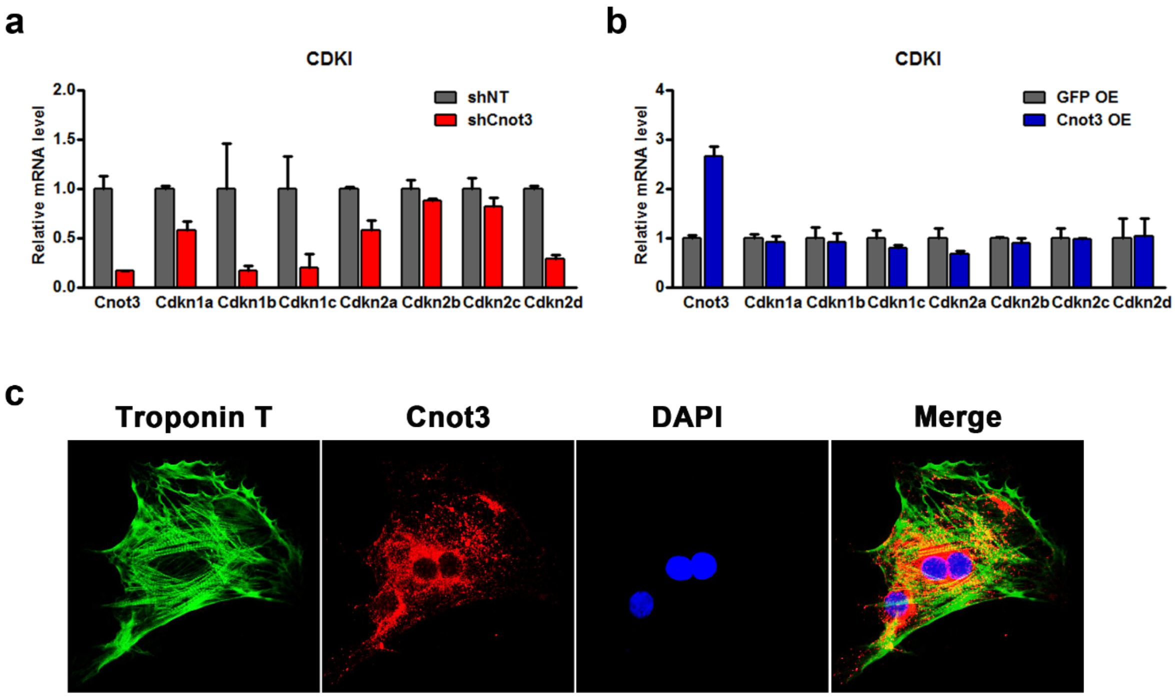

Figure S4

Figure S4. The regulation of Cnot3 in cardiomyocyte proliferation. (a-b) RT-PCR to show the expression of CDKI 96 hours after Cnot3 depletion (a) or expression (b) in human fibroblast. β-actin was used as endogenous control and values were plotted as mean ± SEM from three independent experiments. **(c)** Representative immunofluorescence staining against Tnnt2 (Green) and Cnot3 (Red) to show the localization of Cnot3 in cardiomyocyte. DAPI (blue) was used to stain the cell nuclei. Magnification:200×.

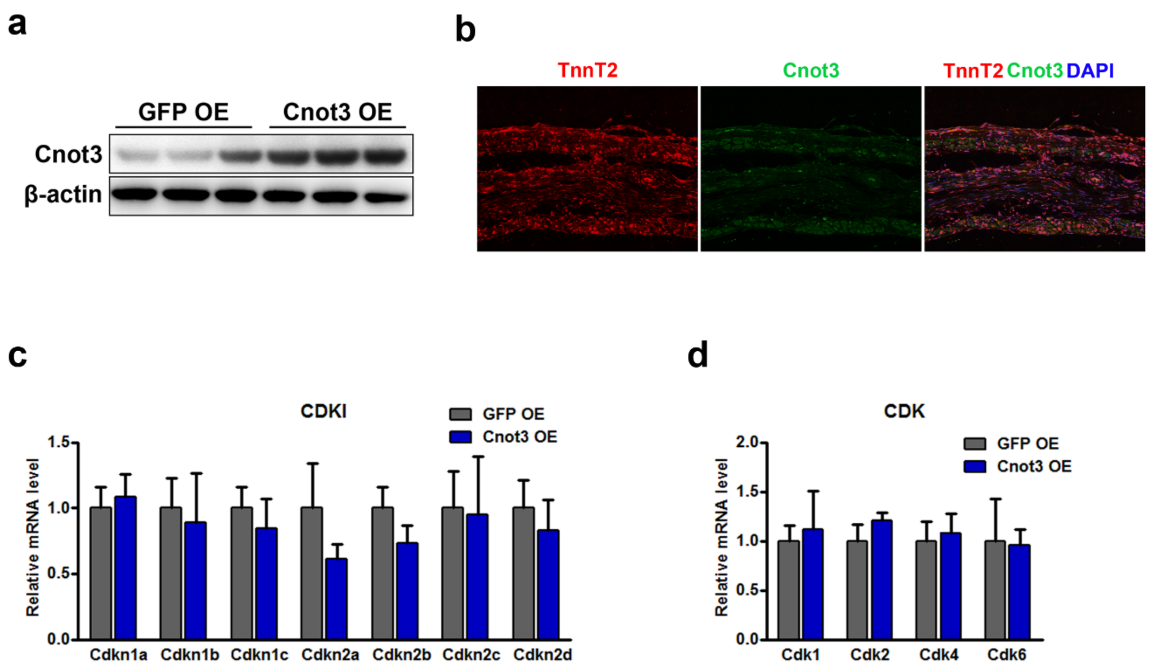

Figure S5

Figure S5. Cnot3 overexpression induces human cardiomyocyte proliferation. (a) Western blot to show successful overexpression of Cnot3 in mouse infarcted hearts. (b) IHC staining against Cnot3 to show the distribution of ectopically expressed Cnot3 in infarcted myocardium. (c-d) Real-time PCR to show the expression of CDKI (c) and CDK (d) in infarcted hearts in response to Cnot3 overexpression.

Table S1. qPCR primers used in the study.

hCnot3 Left	ggacgttccacagacagtga
hCnot3 Right	gagggtgctggttgctgt
hACTN2 Left	cctatccccgggactaagac
hACTN2 Right	aggcagtgctgtcctcttct
hTNNT2 Right	gtcggcagctgctgttct
hTNNT2 Left	tcctctctccagtcctcctct
heIF5 Left	tcgtgtcaaagcagaaccat
heIF5 Right	gcagccttcgaatacacca
hGAPDH Left	ccccggtttctataaattgagc
hGAPDH Right	caccttccccatggtgtct
hβ-actin Left	ccaaccgcgagaagatga
hβ-actin Right	ccagaggcgtacagggatag
hCDKN1A Left	ccgaagtcagttccttgtgg
hCDKN1A Right	catgggttctgacggacat
hCDKN1B Left	actcgccgtgtcaatcattt
hCDKN1B Right	aacaccccgaaaagacgag
hCDKN1C Left	ctcctttccccttcttctcg
hCDKN1C Right	tccatcgtggatgtgctg

hCDKN2A Left	gtggacctggctgaggag
hCDKN2A Right	ctttcaatcggggatgtctg
hCDKN2B Left	gcggggactagtggagaag
hCDKN2B Right	ctgcccatcatcatgacct
hCDKN2C Left	gactatcccttcggcgaga
hCDKN2C Right	aaggctcggccattctttag
hCDKN2D Left	atgacctccagggagccta
hCDKN2D Right	cgtgcacacttcaggtctct
hCDK2 Left	cctcctgggctgcaaata
hCDK2 Right	cagaatctccagggaataggg
hCDK4 Left	gtgcagtcggtggtacctg
hCDK4 Right	ttcgcttgtgtgggttaaaa
hCDK6 Left	tgatcaactaggaaaaatcttgga
hCDK6 Right	ggcaacatctctaggccagt
